# Basal Cell Carcinoma Research Landscape Overview via Latent Dirichlet Allocation and HJ-Biplot Analysis

**DOI:** 10.3390/cancers18142312

**Published:** 2026-07-17

**Authors:** Karime Montes-Escobar, Javier de La Hoz-Maestre, Humberto Llinás-Solano, Carlos Alfredo Salas-Macias, Esteban Fernández-Moreira, Martha Fors, Santiago J. Ballaz

**Affiliations:** 1 Departamento de Ciencias Agrícolas, Facultad de Ingeniería Agrícola, Universidad Técnica de Manabí, Portoviejo 130105, Ecuador; karime.montes@utm.edu.ec; 2 Departamento de Formación y Desarrollo Científico en Ingeniería, Facultad de Ingeniería, Ciencia y Tecnología, Universidad Bernardo O’Higgins, Santiago 8370993, Chile; 3 Facultad de Ingeniería, Universidad del Magdalena, Santa Marta 470004, Colombia; jdelahoz@unimagdalena.edu.co; 4 Departamento de Matemáticas y Estadística, Division de Ciencias Básicas, Universidad del Norte, Barranquilla 080001, Colombia; hllinas@uninorte.edu.co; 5 Facultad de Ingenierías Agroambientales, Universidad Técnica de Manabí, Portoviejo 130105, Ecuador; csalas@utm.edu.ec; 6 Facultad de Medicina, Universidad Complutense of Madrid (UCM), 28040 Madrid, Spain; 7 Faculty of Health Sciences, Universidad Espiritu Santo, Samborondón 092301, Ecuador; 8 Medical School, Universidad de las Américas (UDLA), Quito 170124, Ecuador; martha.fors@udla.edu.ec; 9 Centro de Investigación Biomédica (CENBIO), Facultad de Ciencias de la Salud Eugenio Espejo, Universidad UTE, Quito 170129, Ecuador

**Keywords:** basal cell carcinoma, diagnosis, machine learning, prevention and control, bibliometrics, therapeutics

## Abstract

**Background**: Basal cell carcinoma (BCC) of the skin is the most common cancer in humans, and its incidence rises annually. Despite its low death rate, BCC causes significant morbidity because of its destructive nature to local tissues. The aim of this study was to review the state of the BCC research landscape using data published in Scopus from 1972 to 2023. **Methods**: Using the R package Bibliometrix, we first determined authors, countries, journals, and main topics behind the advancement of BCC research. The Latent Dirichlet Allocation (LDA), a probabilistic topic algorithm, was applied to automatically discover latent (hidden) thematic structures. Additionally, the HJ-Biplot was also chosen to visualize graphical representations of multivariate scientometric and bibliometric data to improve the LDA outcome. **Results**: Over five decades, we discovered 32 unique themes. BCC research has shifted from studying tumor and immunohistochemically characterization, epidemiology, and surgical/histological clearance to the advancement of diagnostic and imaging techniques, like dermoscopy and reflectance confocal microscopy (RCM). Another exciting BCC research trend has been the discovery of aberrant activation within the Hedgehog signaling. Finally, patient treatment, especially surgery, radiotherapy, topical fluorouracil, and imiquimod, is an object of intense research. **Conclusions**: By displaying each topic as a group of related words, this in-depth exploration outlined emerging avenues in BCC evidence-based research that will benefit from the development of cutting-edge diagnostic procedures, as well as a deeper knowledge of BCC etiology and genetic underpinnings, the quality of life in BCC patients after surgery, and the application of artificial intelligence.

## Simple Summary

Basal cell carcinoma (BCC) is the most prevalent skin malignancy globally, causing significant tissue damage despite its low mortality rate. This study analyzes five decades of published research from 1972 to 2023 to help researchers navigate the massive volume of scientific data on this disease. By using a combination of statistical and bibliometric algorithms to scan thousands of articles. We mapped how the scientific focus has shifted over time. We found that research has transitioned from basic tumor descriptions to cutting-edge imaging technologies, genetic discoveries, and targeted therapies. This study provides a comprehensive roadmap of the research landscape, helping the medical community identify current emerging trends and nascent research clusters based on historical momentum. Ultimately, it highlights how future breakthroughs in BCC will likely rely on artificial intelligence, advanced genetics, and improving the quality of life for patients after surgery.

## 1. Introduction

Basal cell carcinoma of the skin or BCC, often grouped with squamous cell carcinoma under the umbrella term of non-melanoma skin cancer, is an essentially indolent, locally destructive skin malignancy and the most common tumor affecting Caucasians [1]. BCC gets its name because it is composed of cells similar to those found in the basal layers of the epidermis and appendages. Anatomical locations where BCCs can arise include the head and neck (80%), trunk, and lower limbs. Cumulative sun exposure and UV radiation are regarded as critical in the pathogenesis of BCC, but the relationship is yet unknown [2]. Independent phenotypic factors, such as light skin color and red hair [3], immunodepression [4], as well as genetic factors, are associated with BCC pathogenesis [5,6].

BCCs incidence rises by 10 million cases per year worldwide. Potential drivers are enhanced diagnosis, aging population, and changes in sun exposure habits [2,7,8,9]. Although eminently curable when the diagnosis is made promptly and the lesion treated in its early phase, metastatic BCC with poor prognosis may occur more often than previously believed [10]. BCC causes significant morbidity because of its destructive nature to local tissues, particularly on the head and neck [11]. This lowers the quality of a patient’s life and places a significant financial burden on the health care system.

The aim of this study was to present a thorough and current overview of the current research trends, nascent research clusters, and underexplored thematic intersections in BCC based on five decades of cumulative data. We used Scopus to carefully plan and execute a systematic search. A meticulously crafted Boolean search string guaranteed thorough retrieval of peer-reviewed articles. The study maintained a high signal-to-noise ratio by eliminating non-relevant documents. Because scientific mappings, bibliometric methods, and network visualization studies [12] fall short in offering text analysis, we adopted a combination of machine learning (Latent Dirichlet Allocation) and multivariate statistical methods (HJ-Biplot) to untangle the BCC research landscape. Additionally, heatmaps helped examine and understand how thematic patterns were related to variables like publication year, country, and journal. By leveraging this dual-methodological approach, we allow the intrinsic statistical properties of literature to generate more granular and pertinent insights than standard bibliometric analysis.

## 2. Materials and Methods

### 2.1. Search Strategy

**Figure 1:**
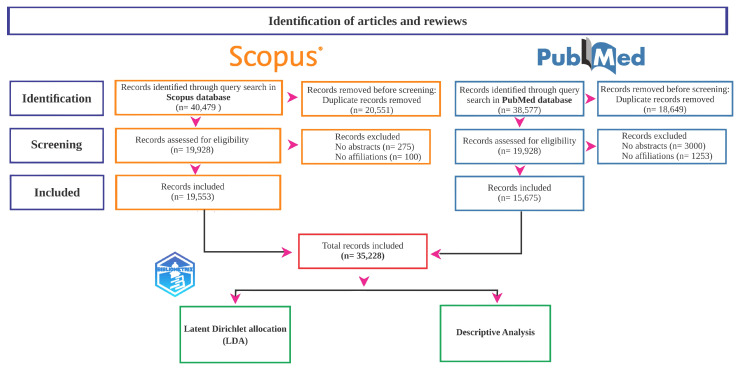
Flow diagram showing the article selection workflow on BCC.

The inclusion criteria were all the research documents, written in English in peer-reviewed journals, that were published in Scopus between 1973 (the year of the earliest BCC research) and 6 October 2023 (a 50-year period), dealing with BCC. To ensure dataset cleanliness/homogeneity, books, book chapters, gray literature, and reports were excluded. The selection of Scopus was a strategic methodological decision, since it is one of the largest and most comprehensive abstract and citation databases of peer-reviewed literature [13,14]. More importantly, Scopus proves superior in terms of coverage in the health sciences field, its indexing accuracy, and its “federated search interface” (i.e., functionality), which allows us to use a common/standardized search form to query the content found across its varied sources necessary for macroscopic scientometric mapping. The following keyword search string was adopted to identify eligible documents: TITLE-ABS-KEY (“basal cell carcinoma”) AND PUBYEAR > 1973 AND PUBYEAR < 2024 AND (LIMIT-TO (DOCTYPE, “ar”) OR LIMIT-TO (DOCTYPE, “re”)). The lead author, in coordination with the co-authors, screened the full texts of all potentially eligible articles. A total of 23,680 out of 28,553 documents that met the inclusion criteria (Figure 1) were compiled and stored in a dataset as CSV files.

### 2.2. Bibliometric Analysis

The following queries were made: (Q1) Who are the key authors and countries behind the advancement of BCC research? (Q2) Which journals act as the core hubs for cutting-edge BCC research? (Q3) How are these research topics distributed across countries and scientific journals? (Q4) In what ways are these research areas evolving? (Q5) What core research areas serve as the conceptual foundation for BCC research? Bibliometric analysis was conducted using the R package Bibliometrix to track published BCC research [15,16]. Three levels of analysis—countries, sources, and authors—were included at this stage to answer Q1, Q2, and Q3.

### 2.3. Latent Dirichlet Allocation (LDA)

To address Q4 and Q5, LDA [17]—an unsupervised, probabilistic topic modeling algorithm—was applied across three distinct phases: preprocessing, LDA model creation, and topic labeling. LDAShiny [18], an open-source R package, was used for the first two phases. During preprocessing, documents were tokenized into individual words, followed by the removal of numbers, single characters, punctuation marks, words occurring only once, and stop words like “the”, “and”, “were”, and “is”. The list of stop words was obtained from standard libraries such as NLTK (i.e., natural language tool kit) and Snowball. Finally, lemmatization normalization was applied to reduce words to their base linguistic forms.

The LDA model was validated using the topic coherence metric, which evaluates the semantic interpretability of generated topics from a human perspective [19]. The score was calculated using the Cv metric of the TextmineR package, which looks at the frequency and co-occurrence of terms inside a topic, indicating improved interpretability. The R package facilitated the optimization of the ideal number of topics (k). To strike a compromise between granularity and thematic clarity, we chose the model with the best coherence score among those in the examined range. The number of topics to be uncovered (the k parameter) varied from 4 to 30, thus creating 26 different LDA models.

#### Assigning Labels to Topics

Because LDA models do not inherently assign semantic labels to discovered topics, and algorithmic approaches possess structural limitations in fully comprehending human language nuances [20], manual annotation remains the standard practice in topic modeling workflows to ensure semantic fidelity [19]. To provide a semantically meaningful and logical interpretation of the probability distributions, topics were manually labeled by carefully examining the top 15 high-probability words, as well as the titles and content of the documents. The manual topic labeling was completed by a diverse team of ten experts, including the authors and independent researchers with backgrounds in oncological research and bibliometric analysis. To validate and summarize the detected topics, the three articles demonstrating the strongest connections to each topic were given to the team based on their thematic coherence and content alignment. In addition to evaluating the body of the current literature to identify research trends, gaps, and landmark publications, they were tasked with independently validating and summarizing the highlighted themes. To minimize bias, improve rigor, and guarantee that the thematic structures and semantic interpretations matched the goals of the study, any interpretative discrepancies among team members were settled via a blind consensus process. Restricting the granular evaluation to three primary articles per topic ensured a highly focused and manageable synopsis of salient features without overburdening the analysis. These foundational texts were distilled into brief summaries that encapsulated the essence of each topic. In addition to offering a gold-standard reference [20], this manual approach guaranteed interpretability and relevance within the context of the study.

To further improve the labeling, the topics were projected into a two-dimensional space by calculating the inter-topic distances [21] and applying multidimensional scaling [22]. This two-dimensional topic representation helped identify similarities between topic labels. The primary outputs of the LDA model consisted of the document-topic and the topic-word distributions. To interpret these findings, we adopted the quantitative indices suggested by Xiong et al. [22] obtained by aggregating the document–topic and topic–word distributions at various levels as follows.

The distribution of topics over time was obtained by

(1) $$\theta_{k}^{y}=\sum_{m\epsilon y}\theta_{mk}/n^{y},$$

where *m* ϵ *y* represents articles published each year, *θ_mk_* the proportion of the *k*-th topic in each item and *n^y^* the total number of articles published in the year.

Topic distribution across journals is defined as the ratio of the *k*-th topic in the journal *j*:

(2) $$\theta_{k}^{j}=\sum_{m\epsilon j}\theta_{mk}/n^{j},$$

where *m* ϵ *j* represents the articles in a particular journal, *θ_mk_* the proportion of the *k*-th topic on each item, and *n^j^* the total number of articles published in the journal *j*.

Topic distribution across countries is defined as the ratio of the *k*-th topic in the country *c*:

(3) $$\theta_{k}^{c}=\sum_{m\epsilon c}\theta_{mk}/n^{c},$$

where *m* ϵ *c* represents the articles in a particular country, *θ_mk_* the proportion of the *k*-th topic on each item, and *n^c^* the total number of articles published in the country *c*.

### 2.4. Statistics

In terms of tendency characterization, we used simple regression slopes for each topic where the publication year served as the independent variable, and the annual proportion of the topic functioned as the dependent (response) variable [23]. We determined the directionality of these trends (positive slopes = upward trends; negative slopes = declining trends) and set a significance threshold of *p* < 0.01.

### 2.5. HJ-Biplot

Like a multivariate extension of standard scatter plots, biplot factorizations introduced by Gabriel [24], such as the GH-Biplot and JK-Biplot, are graphical representations of multivariate data that can visualize three or more variables. The HJ-Biplot [25] is a low-dimensional space alternative to the standard biplots that simultaneously maximizes the representation quality of both rows (years, journals, or countries) and columns (topics) in a shared low-dimensional space. To maximize representation quality, the underlying data matrix was subjected to singular value decomposition (SVD), which projects rows and columns as vectors onto a two-dimensional plane. The weighted frequencies of keywords across documents were used to determine the likelihood they will appear. By normalizing each keyword’s frequency within a topic in relation to its overall presence across all topics, the contribution of each keyword to a topic was calculated. This normalization ensured that the probabilities reflected the keyword’s significance to specific topics, enabling accurate multivariate analysis.

The data visualization involved representing a multivariate data matrix $X_{nxp}$ using two sets of vectors: markers $g_{1}$, $g_{2},\ldots ,\;g_{n}$ for rows and markers $h_{1}$, $h_{2},\ldots ,\;h_{p}$ for columns. Each row corresponded to a subject, and each column to a variable, allowing both marker sets to be projected onto a shared reference system with optimal representation quality. With matrix A defining markers $g_{1}$, $g_{2},\ldots ,\;g_{n}$ and matrix B defining markers $h_{1}$, $h_{2},\ldots ,\;h_{p}$, the relationship was expressed as $X=AB^{T}$.

The markers were derived through the singular value decomposition (SVD) of the data matrix X, expressed as ${X=UDV}^{T}.\;$ Here, *U* contains the eigenvectors of ${XX}^{T}$, V holds the eigenvectors of $X^{T}X$, and D is a diagonal matrix of the singular values $\sigma_{i}$ of X. Matrices A and B correspond to the first two columns of UD and VD, respectively.

The HJ-Biplot was used as a complementary analytical tool to enhance and enrich the findings of the LDA model [26,27,28]. Interpreting the HJ-Biplot combines guidelines from multiple techniques, including multidimensional scaling, correspondence analysis, factor analysis, and classic principal component biplots [25]. All computational workflows and visual projections at this stage of the analysis were executed using the Multbiplot software (version 25.11.15) package [29].

## 3. Results

Most of the BCC information published from 1973 to 2023 was sourced from 3625 journals (Table 1). The noteworthy yearly growth rate of 4.73% indicated a steady and continuous publication. The document’s average age of 15.2 years reflected a well-balanced combination of contributions and contemporary insights from a historical perspective. Its impact and recognition were attested to by an average count of 32.03 cites per document. In terms of document types, the collection was characterized by a prevalence of articles (19,770) and reviews (3910), emphasizing the primary formats shaping the discourse in BCC literature. This wealth of information emphasizes the primary formats shaping the scientific discourse on BCC over the 50-year study period.

A detailed examination through keyword analysis unveiled a substantial lexicon exceeding 44,000 unique terms. This thorough analysis contributes to a nuanced and comprehensive understanding of the multifaceted aspects associated with BCC (Table 1). Strong collaborative dynamics were evident in the involvement of 77,002 authors, with a collaboration rate of 5.39 co-authors per document. This signifies a dynamic and diverse collaboration landscape in the exploration of BCC International collaboration, constituting 14.24% of co-authorships (Table 1).

**Figure 2:**
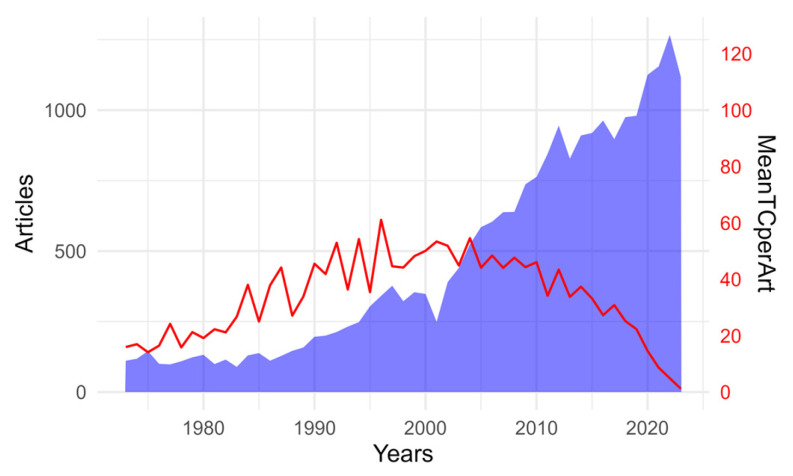
Temporal analysis of citations and number of articles in BCC research.

The number of articles about BCC has steadily increased over the 50-year study period, and the scientific output shows a strong upward trajectory (Figure 2). It implies ongoing interest in and expansion of this field’s study. The average number of citations per article exhibits a fluctuating temporal dynamic. Particularly, the years 1992 and 2006 exhibited significant peaks in the mean numbers of citations, signifying periods of noteworthy impact and attention within the scientific literature (Figure 2). The 1990s and early 2000s emerged as periods of intensified activity in terms of citations and article production. This surge is likely attributable to landmark breakthroughs in the understanding of BCC during that period. There has, however, been a downward trend in the mean of citations per publication since the 2010s. This phenomenon may indicate a shift in research preferences, the introduction of new approaches, or a reflection of inherent bibliometric citation lag, as recent publications have had less time to accumulate citations. Despite the decrease in mean citations, the total number of articles has experienced a significant increase in recent years, reaching a peak in 2021. This implies that the scientific output on BCC is still growing.

### 3.1. Sources

Table 2 provides a comprehensive overview of the most influential journals on Dermatology between 1973 and December 2023. The key metrics include the h-index (reflecting a journal’s joint productivity and citation impact), TC (total citations), NP (number of publications), and PY_start (initial publication year). The *Journal of the American Academy of Dermatology* leads the cohort and stands out for having an elevated h-index of 97, 570 publications and 37,136 total citations since its beginning in 1980. The *British Journal of Dermatology* follows as the second influential journal, having garnered attention with 523 publications, 30,668 total citations, and a h-index of 90, showcasing its enduring influence since 1973. With 475 publications since 1995, *Dermatologic Surgery* ranked third. The journal has a total of 13,455 citations and an h-index of 59. Rounding the top fourth position was the *Journal of Cutaneous Pathology*, which has made significant contributions by publishing 345 articles and accumulating 9208 total citations since 1974. Its noteworthy h-index is 49.

### 3.2. Authors

Table 3 provides a detailed overview of the top ten most prolific authors in BCC research, drawing insights from the analysis of 23,680 articles published between 1973 and 2023. This compilation offers a comprehensive overview of these influential authors, illuminating key bibliometric indicators such as their impact, citation counts, publication output, and the long-term significance of their contributions to the field. Leading the cohort is Argenziano G with an impressive h-index of 33, accumulating 4,195 total citations from 85 publications since 1974. Han J, who has a h-index of 32, 4007 citations, and 72 publications starting from 2004, follows in the second position.

### 3.3. Countries

**Figure 3:**
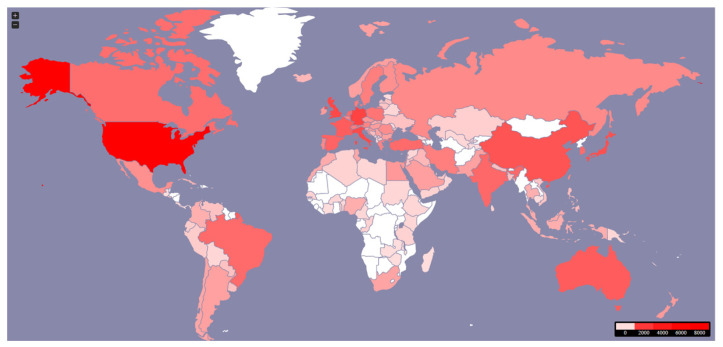
Word map showing the distribution of countries that contributed to the 23,680 articles published about BCC between 1973 and 2023.

A world map (Figure 3) reveals the research conducted on BCC in 116 countries on every continent. Bibliometric data extracted for the top 30 most productive nations (Table 4) delineates prominent spatial patterns in scientific output. The United States occupies the preeminent position globally, leading in both absolute publication volume (NP = 6688) and citations (TC = 210,348). This results in an average article citation of 49.5. Germany and the United Kingdom are notable contributors to the global BCC research corpus, with 1580 and 1360 publications, respectively. The discernible prominence of Switzerland is noteworthy, as seen by its exceptionally elevated average article citation of 51.5. This data implies that publications originating from this jurisdiction received more attention from the scientific community. Sweden is not far behind, demonstrating the impactful efforts of its BCC research with an average citation of 54.4. In terms of publication volume, the analysis also revealed a diverse and international representation, with conspicuous contributions from countries such as China and India. However, it is discernible that these nations exhibit comparatively diminished average article citations, indicative of potential areas meriting further opportunities for enhanced citation visibility and international collaborative partnerships. Focusing on average article citations, Finland commands attention with a remarkably elevated value of 56.2, indicating a concentration of impactful BCC research within its borders. Conversely, countries such as Mexico and Egypt register lower average article citations, signaling prospects for advancement and heightened visibility within the BCC field. These findings underscore the expansive global purview of BCC research, elucidating both the quantitative and qualitative dimensions of contributions emanating from diverse nations. The insights derived from this analysis hold substantive value for researchers, policymakers, and healthcare professionals engaged in the BCC domain.

### 3.4. Latent Dirichlet Allocation

The LDA model, characterized by the optimal coherence score, encompasses a total of 22 topics (k = 22). Table 5 provides a summary of the 22 most probable terms, meaning that these are the terms with the highest probabilities for each latent topic, along with their semantically related labels.

### 3.5. Topic Trends

**Figure 4:**
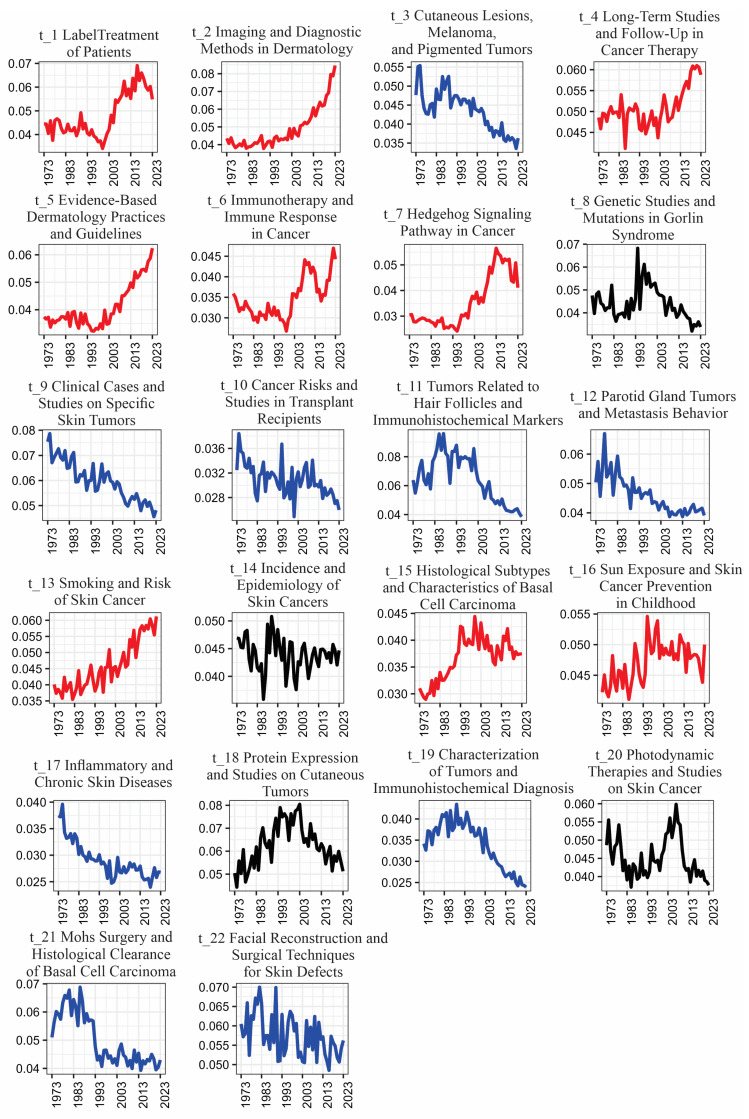
Trends of research topics in BCC between 1973 and 2023: increasing (red), decreasing (blue), and stable (black) topic dynamics over time.

The topic distribution by document $\theta_{m}$ was added to compute the average probability $\theta_{k}^{y}$ of all the articles published in a particular year to identify the trends (Figure 4). We found that the probabilities of some topics demonstrating a steady and statistically significant expansion over time (red), topics exhibiting a contracting or downward trajectory (blue), and stable topics with no discernible long-term trend (black).

### 3.6. Topic Distribution over Publication Sources

**Figure 5:**
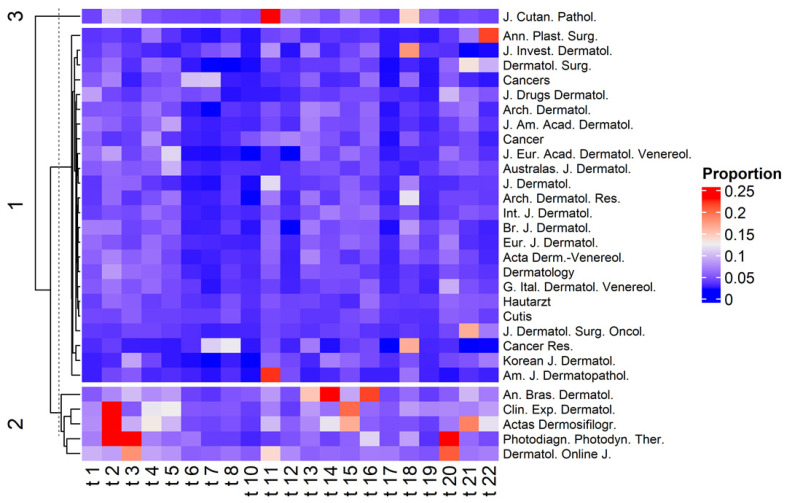
Heatmap displaying the proportional topic in the top 30 analyzed sources. The dotted cut-off line establishes the similarity threshold in the dendrogram to group the journals into three distinct clusters (1, 2, and 3) based on the proportion of topics they share over time.

In this section, topic distributions within publication sources were computed using Equation (2) and visually represented by means of a heatmap (Figure 5). Research topics were arranged in columns, publication sources in rows, to determine the likelihood they will appear in the sources. In addition, the topic distributions are arranged in the publication sources like a matrix. The Euclidean distance metric was applied to the topic probability profiles to calculate the geometric proximity between the different publication sources. The resulting distance matrix revealed distinct patterns, justifying the application of unsupervised clustering methods. The next step was to perform hierarchical clustering using the average-linkage method. The outcomes of this clustering process are depicted in the clustering dendrograms on the left side of Figure 5. These results provide insights into the focus areas of each publication source, the categorization of topics sharing commonalities, and the identification of sources that tend to concentrate on studies of a similar nature.

### 3.7. Topic Distribution Among Countries of Publication

**Figure 6:**
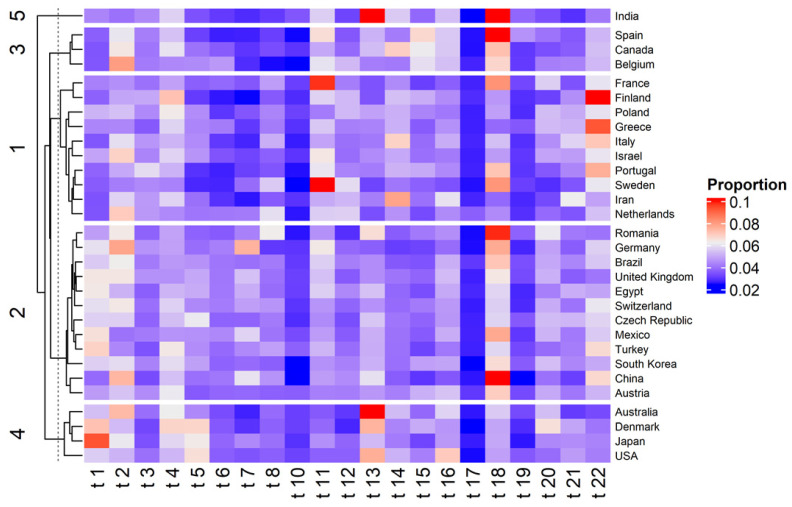
Heatmap overview of the proportional topic in the top 30 countries analyzed. The dotted cut-off line establishes the similarity threshold in the dendrogram to group the journals into three distinct clusters (1, 2, and 3) based on the proportion of topics they share over time.

Heatmaps helped us understand how thematic patterns were related to variables like country and journals. Red highlighting indicates the strongest associations among variables, reflecting elevated probability densities. The 30 countries with the highest publication volume were selected based on the analysis done at the publication sources level in Section 3.6. The clustering heatmap depicted in Figure 6 was created by using the aggregation formula presented in Equation (3) of this study. According to the BCC study, most countries displayed a relatively dispersed distribution of research topics without being restricted to a few particular subjects. Nonetheless, some countries continue to focus on specific research areas. Utilizing the methodology presented in the preceding sections, this part does a clustering analysis on the topic distribution across countries. Notably, countries in a cluster category do not always exhibit geographic proximity, thus suggesting that cooperation between countries extends beyond their geographical boundaries. Examining the topic distribution in the groups shows that countries share a common focus on areas of study. For instance, Australia and India have the same interest in t_13 (Smoking and Risk of Skin Cancer). In France, Brazil, Romania, Chile, and Mexico, the focus is placed on t_18 (Protein Expression and Studies on Cutaneous Tumors) (Figure 6). LDA algorithm’s identification of the core BCC research latent themes was mirrored in the relevant clustergrams that emerged from the hierarchical cluster analysis of the heatmaps.

**Figure 7:**
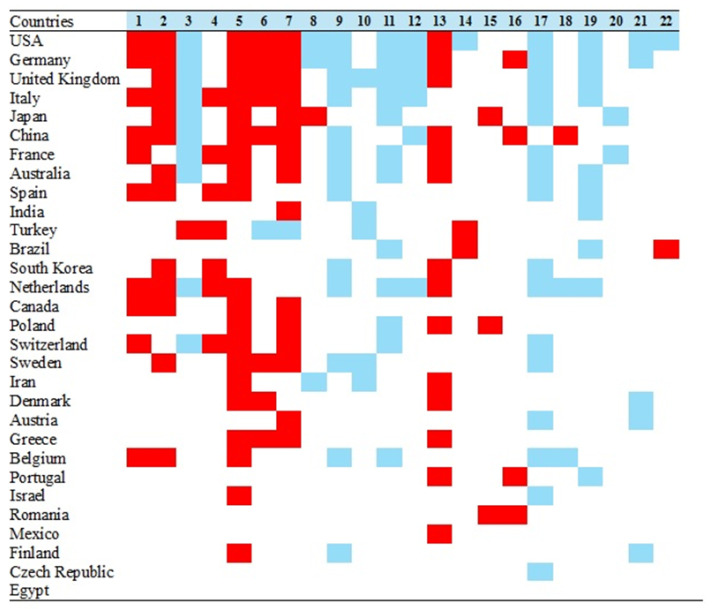
Topic trends research in BCC by countries. Red color indicates increasing tendency, blue decreasing tendency, and white fluctuating or no prominent trends.

To understand the differences among countries, we investigated how the topic distribution evolved over time across those countries. Overall, as indicated in Figure 7, topic t_5 (Evidence-Based Dermatology Practices and Guidelines) showed a positive trend in most of the evaluated countries (20 out of 30). In contrast, topics t_9 (Clinical Cases and Studies on Specific Skin Tumors), t_10 (Cancer Risks and Studies in Transplant Recipients), and t_11(Tumors Related to Hair Follicles and Immunohistochemical Markers) for the evaluated countries displayed either a negative trend, fluctuating trends, or no prominent trends at all.

### 3.8. HJ-Biplot Analysis

**Figure 8:**
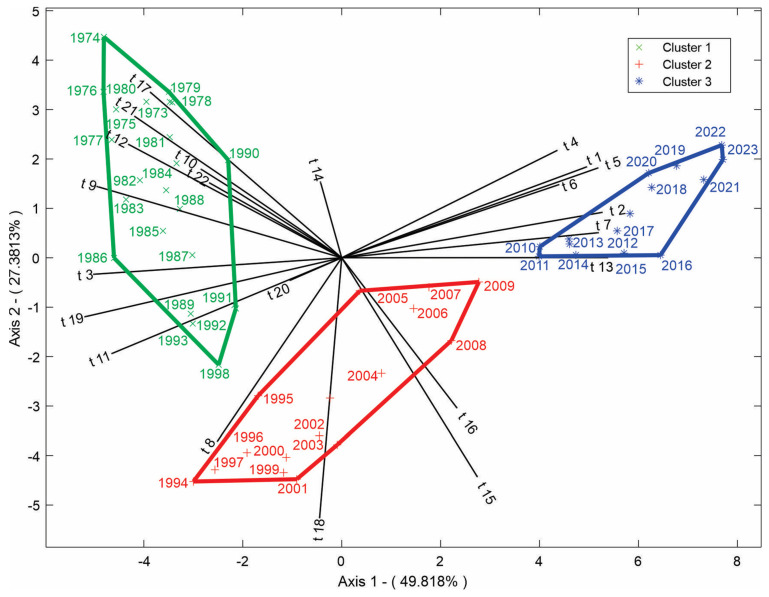
Association among topics per year using the HJ-Biplot method. Vector angles (Cosine) represent their correlation. Vector length represents the variance and discriminatory power of that specific topic within the dataset. The proximity between row points (countries/journals, etc.) indicates similarities in their thematic profiles.

**Figure 9:**
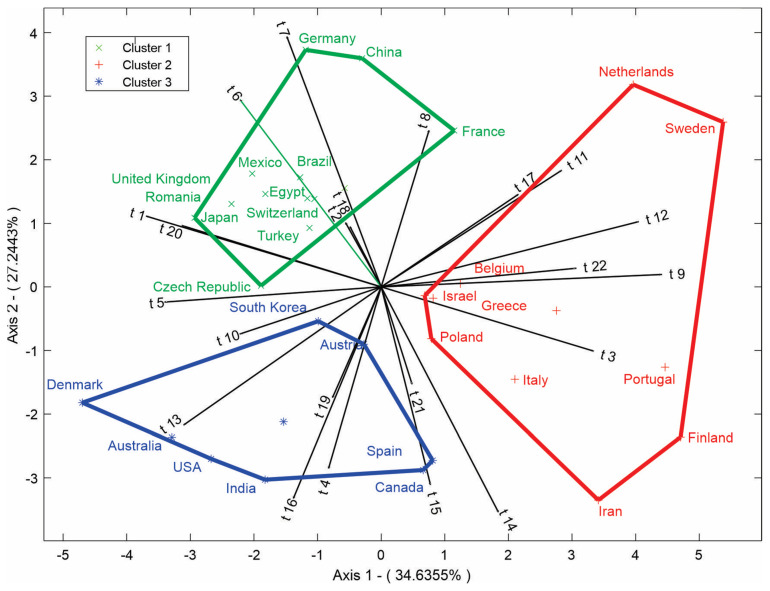
Association among topics per country using the HJ-Biplot method. Vector angles (Cosine) represent their correlation. Vector length represents the variance and discriminatory power of that specific topic within the dataset. The proximity between row points (countries/journals, etc.) indicates similarities in their thematic profiles.

**Figure 10:**
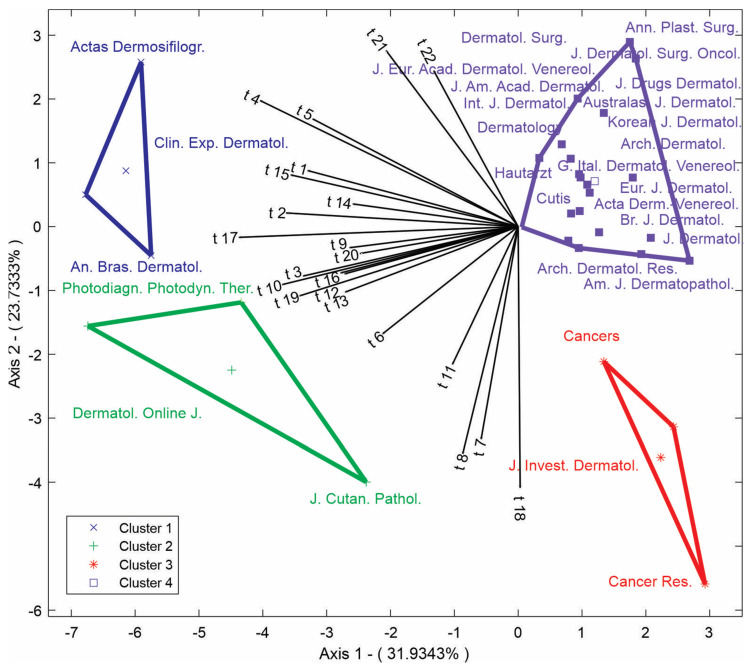
Association among topics per journals using the HJ-Biplot method. Vector angles (Cosine) represent their correlation. Vector length represents the variance and discriminatory power of that specific topic within the dataset. The proximity between row points (countries/journals, etc.) indicates similarities in their thematic profiles.

The examination was carried out across temporal, periodical, and geographical dimensions using multivariate HJ-Biplot analysis, which yielded the latent topic distribution (theta) matrix outcomes. The probability coefficients of each resultant matrix ranged from 0 to 1. As shown in Figure 8, Figure 9 and Figure 10, the two-dimensional biplot projections capture a high percentage of cumulative explained variance across all three analytical dimensions. Specifically, the first two axes account for 77.19% of the total data variability for the topic-year intersection (Figure 8), 61.87% for the topic–country intersection (Figure 9), and 55.66% for the topic-journal intersection (Figure 10).

The topics that were more pertinent between 1973 and 1998 were the following: t_20 (Photodynamic Therapies and Studies on Skin Cancer), t_11 (Tumors Related to Hair Follicles and Immunohistochemical Markers), t_19 (Characterization of Tumors and Immunohistochemical Diagnosis), t_3 (Cutaneous Lesions, Melanoma, and Pigmented Tumors), t_9 (Clinical Cases and Studies on Specific Skin Tumors), t_12 (Parotid Gland Tumors and Metastasis Behavior), t_22 (Facial Reconstruction and Surgical Techniques for Skin Defects), t_10 (Cancer Risks and Studies in Transplant Recipients), t_21 (Mohs Surgery and Histological Clearance of Basal Cell Carcinoma), t_17 (Inflammatory and Chronic Skin Diseases) and t_14 (Incidence and Epidemiology of Skin Cancers). The topics for 1999 through 2009 were: t_8 (Genetic Studies and Mutations in Gorlin Syndrome), t_18 (Protein Expression and Studies on Cutaneous Tumors), t_15 (Histological Subtypes and Characteristics of Basal Cell Carcinoma), and t_16 (Sun Exposure and Skin Cancer Prevention in Childhood). Finally, the most relevant topics for the years 2010–2023 were: t_13 (Smoking and Risk of Skin Cancer), t_7 (Hedgehog Signaling Pathway in Cancer), t_2 (Imaging and Diagnostic Methods in Dermatology), t_6 (Immunotherapy and Immune Response in Cancer), t_5 (Evidence-Based Dermatology Practices and Guidelines), t_1 (Treatment of Patients), and t_4 (Long-Term Studies and Follow-Up in Cancer Therapy). The information mentioned above is graphically depicted in Figure 8.

The connections between the topics and countries where the information is generated can be classified into three groups (Figure 9). In the first group, countries like Germany, China, France, Brazil, Mexico, United Kingdom, Romania, Egypt, Japan, Switzerland, Turkey and, Czech Republic are associated with topics t_8 (Genetic Studies and Mutations in Gorlin Syndrome), t_7 (Hedgehog Signaling Pathway in Cancer), t_18 (Protein Expression and Studies on Cutaneous Tumors), t_2 (Imaging and Diagnostic Methods in Dermatology), t6 (Immunotherapy and Immune Response in Cancer), t_1 (Treatment of Patients), t_20 (Photodynamic Therapies and Studies on Skin Cancer) and, t_5 (Evidence-Based Dermatology Practices and Guidelines). The second group including South Korea, Austria, Denmark, Australia, USA, India, Canada, and Spain is related to topics t_10 (Cancer Risks and Studies in Transplant Recipients), t_13 (Smoking and Risk of Skin Cancer), t_19 (Characterization of Tumors and Immunohistochemical Diagnosis), t16 (Sun Exposure and Skin Cancer Prevention in Childhood), t_4 (Long-Term Studies and Follow-Up in Cancer Therapy), t_15 (Histological Subtypes and Characteristics of Basal Cell Carcinoma), t_21 (Mohs Surgery and Histological Clearance of Basal Cell Carcinoma), and t_14 (Incidence and Epidemiology of Skin Cancers). The final group of countries links Iran, Finland, Portugal, Italy, Poland, Greece, Israel, Belgium, Sweden, and Netherlands to the following topics: t_7 (Hedgehog Signaling Pathway in Cancer), t_11(Tumors Related to Hair Follicles and Immunohistochemical Markers), t_12 (Parotid Gland Tumors and Metastasis Behavior), t_22 (Facial Reconstruction and Surgical Techniques for Skin Defects), t_9 (Clinical Cases and Studies on Specific Skin Tumors) and t_3 (Cutaneous Lesions, Melanoma, and Pigmented Tumors).

In Figure 10, the relationship between topics and journals ultimately reveals four groupings. The following journals in the first group do not directly relate to any topic: *Dermatol. Surg.*, *Ann. Plast. Surg.*, *J. Dermatol. Surg. Oncol.*, *J. Eur. Acad. Dermatol. Venereol.*, *J. Am. Acad. Dermatol.*, *J. Drugs Dermatol.*, *Int. J. Dermatol.*, *Australas. J. Dermatol.*, *Dermatology*, *Korean J. Dermatol.*, *Arch. Dermatol.*, *G. Ital. Dermatol. Venereol.*, *Hautarzt*, *Cutis*, *Eur. J. Dermatol.*, *Acta Derm. Venereol.*, *Br. J. Dermatol.*, *J. Dermatol.*, *Arch. Dermatol. Res.*, and *Am. J. Dermatopathol*. The second group does not associate the topics with the journals *Cancers*, *J. Invest. Dermatol.,* and *Cancer Res*. Regarding group three, it was noted that the journals *J. Cutan. Pathol.*, *Dermatol. Online J.*, and *Photodiagn. Photodyn. Ther.* were connected to the topics t_9 (Clinical Cases and Studies on Specific Skin Tumors), t_20 (Photodynamic Therapies and Studies on Skin Cancer), t_3 (Cutaneous Lesions, Melanoma, and Pigmented Tumors), t_10 (Cancer Risks and Studies in Transplant Recipients), t_16 (Sun Exposure and Skin Cancer Prevention in Childhood), t_9 (Clinical Cases and Studies on Specific Skin Tumors), t_12 (Parotid Gland Tumors and Metastasis Behavior), t_13 (Smoking and Risk of Skin Cancer), t_6 (Immunotherapy and Immune Response in Cancer), t_11 (Tumors Related to Hair Follicles and Immunohistochemical Markers), t_8 (Genetic Studies and Mutations in Gorlin Syndrome), t_7 (Hedgehog Signaling Pathway in Cancer), and t_18 (Protein Expression and Studies on Cutaneous Tumors). The journals *An. Bras. Dermatol.*, *Clin. Exp. Dermatol.*, and *Actas Dermosifilogr.* compromise the fourth group and are associated with the topics t_17 (Inflammatory and Chronic Skin Diseases), t_2 (Imaging and Diagnostic Methods in Dermatology), t_14 (Incidence and Epidemiology of Skin Cancers), t_15 (Histological Subtypes and Characteristics of Basal Cell Carcinoma), t_1 (Treatment of Patients), t_4 (Long-Term Studies and Follow-Up in Cancer Therapy), t_5 (Evidence-Based Dermatology Practices and Guidelines), t_21 (Mohs Surgery and Histological Clearance of Basal Cell Carcinoma), and t_22 (Facial Reconstruction and Surgical Techniques for Skin Defects).

## 4. Discussion

Our scientometric analysis, which spans nearly five decades (1973–2023) delved deep into the landscape of BCC research and discovered 22 unique themes with high coherence scores. This automated, data-driven framework offers unprecedented scalability for massive text datasets while minimizing subjective selection bias. By displaying links across journals, years, countries, and topics in a common low-dimensional space, HJ-Biplot statistics mapped total structural variance rather than volume and successfully isolated emerging fields and historical thematic blind spots. Accuracy, reproducibility, and methodological rigor were guaranteed using the R-based bibliometrix package (version 3.0.5) and advanced Textminer (version 3.0.5) tools.

Document production on BCC research has been growing at an average yearly rate of 4.73%, which underscores a continued interest and expansion in BCC investigation (Figure 2). The yearly increase in BCC incidence, which presents ongoing public health challenges, significant patient morbidity, and escalating healthcare costs, explains why there is such a considerable interest [30,31]. Notable peaks in mean citations for specific years, such as 1992 and 2006, indicated periods of heightened attention and influence, possibly related to significant advances in our understanding of BCC during those years. While a downward trend in mean citations is observable from the 2010s onward, this pattern does not reflect a decline in thematic relevance or a saturation of interest. Rather, as supported by the simultaneously skyrocketing absolute volume of recent publications, this trend is a classic indicator of bibliometric citation lag, combined with a highly distributed modern research landscape where citations are spread across a rapidly expanding volume of specialized venues [32,33]. Ultimately, BCC remains a critical focus of international public health concern and clinical investigation [34].

The USA has the largest number of articles and the total number of publications worldwide, followed by Germany and the United Kingdom. From a total of 23,680 articles concerning BCC, 41.0% were published in these nations. The following countries, Italy, Japan, and China, constituted 15.0% of the total publication counts. Italy had an average of 27 citations per article, although this remains substantially lower than the USA. The USA ranked first in the total number of citations (210,348), followed by the United Kingdom (42,624) and Germany (33,006). After Finland (56.2), Sweden (54.4), Switzerland (51.5), the USA (49.5), Denmark (49.2), and Germany (48.7) lead the average article citations. Among countries/regions, the USA, the United Kingdom, and Germany demonstrated the highest levels of centrality and quality of research on BCC. The analysis of international collaborations and geographic distribution of BCC research provides insights into the leading countries in this field, paving the way for multicentric clinical trials and scientific cooperation.

On an individual level, the latent research themes identified by the LDA model are deeply anchored by the empirical breakthroughs of the field’s most prolific investigators, including Giovanni Pellacani, Jiali Han, and Iris Zalaudek, who have worked on a groundbreaking publication in the last 20 years. Giovanni Pellacani’s work focused on the application of the most popular non-invasive, high-resolution imaging modalities, the dermoscopy and the reflectance confocal microscopy or RCM [35]. In vivo discrimination of skin lesions, allowing benign structures to be confidently spared from unnecessary biopsy while accelerating the excision of malignant presentations, has been facilitated by the combination of RCM imaging and dermoscopic examination [36]. Another noteworthy contribution came from Jiali Han, who identified new areas of human genomes linked to skin cancer risk [9,37,38] and skin cancer epidemiology [39]. Finally, Iris Zalaudek discovered dermoscopic subpatterns of nonpigmented skin tumors [40,41]. These experts’ particular subsets of research were representative of the BCC field’s extraordinary amplitude and evolution.

The discerning analysis of topic trends reveals how various themes have evolved over time, providing valuable insights into the trajectory of BCC research (Figure 4). In the past 13 years, BCC research has shifted from studying tumor and immunohistochemically characterization, epidemiology, and surgical/histological clearance margins to investigating new treatments, long-term skin cancer, medical imaging/dermoscopic diagnosis, the Hedgehog signaling, and smoking’s risks [42,43]. Research topics that have persisted throughout time are the classification of BCC histological subgroups and the dangers of sun exposure, mainly during childhood, for skin cancer. This distribution pattern reflects a deliberate stratification of scientific dissemination in the most prestigious journals (H-index between 71 and 97) such as the Journal of the American Academy of Dermatology, British Journal of Dermatology, and Archives of Dermatology. Recent years have seen the emergence of new themes in BCC, which have been published in journals with substantially lower H indices (between 12 and 28), like the Anais Brasileiros de Dermatologia, Clinical and Experimental Dermatology, and Dermatology Online Journal. For a variety of reasons, top researchers actively utilize these lower-tier, practitioner-focused regional publications to target localized clinical communities and specialized healthcare providers. BCC research trends published in the bottom quartiles attest to the fact that some of the well-known countries in the field of BCC, including Germany, the United Kingdom, China, Japan, Finland, and Switzerland, demonstrate a higher interest in publishing in low-impact journals. Conversely, the USA has concentrated on the majority of conventional topics like histopathological and immunological characterization of BCC subtypes, surgical removal of skin tumors, and incidence/epidemiology of skin cancer. These topics, distributed across journals (Figure 5) and countries (Figure 6 and Figure 7), showcase the dynamic nature of scientific inquiry in the BCC field.

To provide a deeper contextualization of the latent thematic structures identified by the LDA algorithm, a multivariate HJ-Biplot analysis was executed across temporal, periodical, and geographical axes. The longitudinal mapping of topics maps the historical trajectory (Figure 8), transitioning from classical histopathology (t_11, t_19, and t_21) to precision oncology and digital diagnostics (t_2, t_5, and t_7). The topic–country intersection (Figure 9) demonstrates that the global output in BCC research was driven by distinct geopolitical driving forces. Group 1 (Germany, China, France, and the UK) maintains a strong association with advanced therapeutics (t_6 and t_7) and genomic research (t_8) that is heavily underpinned by biotechnology funding, academic medical networks, and robust infrastructural support. Group 2 (the USA, Australia, Spain, Canada, South Korea, and Austria) leads global research in prevention, early screening, and optimized surgical excision (t_4, t_14, t_16, and t_21). Group 3 (Italy, Iran, Finland, Poland, and Greece) reflects strong, region-specific clinical case-profiling (t_9 and t_12) and surgical techniques (t_22). Regarding the academic dissemination ecosystem, Figure 10 outlines how highly specialized thematic pipelines (t_2, t_5, t_20, and t_21) exist in for instance *Photodiagnosis and Photodynamic Therapy*, *Dermo-Sifiliográficas*, *Clinical and Experimental Dermatology* (Groups 3 and 4), whereas high-impact, broad-spectrum dermatological and oncological journals (e.g., *Journal of the American Academy of Dermatology*, *British Journal of Dermatology*, *Dermatologic Surgery*, and *Cancers*) do not tightly align with isolated latent topics.

Clinically, BCCs exhibit several markedly different clinical and histological variants. The superficial variant often mimics benign inflammatory dermatoses such as localized eczema or plaque psoriasis. Other variants include nodular/cystic, superficial, morphoeic (sclerosing), and pigmented [3,5]. The prognosis and disease burden are highly dependent on the BCC subtype and disease stage at diagnosis [44]. As they advance, BCCs can have a wide variety of patterns, which may make classification difficult. While the superficial BCC grows quite slowly, the aggressive morphoeic subtype, which accounts for 5–10% of all BCC, carries a worse prognosis given its delayed presentation, local tissue destruction, tumor recurrence, metastasis, and challenging diagnosis [45]. Hence, the advancement of diagnostic and imaging techniques (Table 5, t_2) for an accurate histologic classification of BCC based on histologic growth patterns constitutes a prominent trend in BCC research [46].

The direct epidemiological evidence that links cumulative sun exposure to BCC is very weak when it comes to the etiology and risk factors of BCC. That certain hereditary disorders predispose to an early onset of BCCs points to a genetic susceptibility [47] that involves the Hedgehog pathway (*PTCH1*, *PTCH2*, *SMO*, and *SUFU* genes) [6]. Exciting advancements in our understanding of BCC biology and its application in the clinic have been made possible by the discovery of the aberrant activation within the Hedgehog signaling, which is a pathognomonic feature of BCC development (Table 5, t_7) [1,48,49]. Long-ignored genes include the tumor suppressor TP53 [50] and the melanocortin-1 receptor (MC1R) [51], which might contribute to the development of sporadic BCC.

The treatment of patients (Table 4, t_1) remains the most prevalent and exciting topic in BCC research, given the increasing prevalence and cost burden of the condition. The most common treatment for BCC is surgery at an early stage, radiotherapy, as well as topical fluorouracil and imiquimod. Photodynamic therapy and carbon dioxide laser are further forms of destructive therapy that are currently evolving. Since 2012, novel (molecular) therapies have been changing the treatment paradigm for advanced BCC. Hedgehog signaling pathway inhibitors are indicated for both the treatment of metastatic BCC and locally advanced BCC that either recurred after surgery or are not amenable to surgery or radiation therapy [11]. Furthermore, immune checkpoint inhibitors, specifically anti-programmed cell death-1 (anti-PD-1) monoclonal antibodies, are actively expanding the therapeutic arsenal for treatment-resistant BCC, adapting oncology frameworks successfully pioneered in other highly immunogenic skin cancers, such as advanced melanoma and Merkel cell carcinoma [52]. Lately, BCC prevention is still a cause of worry. There is a clear association between sun exposure, sunburn in childhood (Table 4, t_16), and smoking (Table 4, t_13) and an increased risk for skin cancer [47]. The BCC evidence-based dermatology (Table 5, t_5) offers valuable insights for researchers, policymakers, and healthcare professionals engaged in the BCC domain [43,53].

Based on five decades of BCC research, this study also untapped potential topics by identifying unexplored areas. One was the study of patient outcomes and quality of life in surgically treated BCC [54]. Another was the integration of artificial intelligence (AI) and deep-learning computer vision models [55]. We also highlight that while dermoscopy and RCM dominate the historical volume of the literature, high-frequency ultrasound (HFUS) [56] represents a critical, expanding frontier—particularly for non-invasive depth mapping. Finally, the last 30 years have seen sporadic publication of evidence supporting the genetic basis for BCC [50,51].

The generative LDA approach offers a level of semantic granularity that standard discriminative or descriptive text-mining models cannot replicate. While co-citation networks map explicit intellectual lineages based on referencing behavior, LDA uncovers latent semantic structures independent of citation counts. Unlike standard keyword lists, LDA extracts probabilistic themes by modeling the conditional probability distributions of words across the entire corpus, offering a more detailed knowledge of topic relationships and structure.

The subjectivity inherent in the manual labeling of LDA topics, the potential for missed publications by using only Scopus, the linguistic bias introduced by excluding non-English articles, the constraints of the “bag-of-words” model, which processes tokens based entirely on co-occurrence frequencies while ignoring grammatical syntax and narrative context, and the impact of excluding literature like reviews and book chapters are some limitations of the study. Although Scopus captures most of the high-impact BCC research, some niche or regional biomedical papers indexed exclusively in Embase, Web of Science, or PubMed may not be present. While LDA can disclose “unknown unknowns”, give an overview of the research landscape to identify new topics and their interconnections, and demonstrate how old topics are emerging in new ones, human intervention is required to manually label these subjects, which may introduce some distortion into the results. This risk was specifically reduced by incorporating the HJ-Biplot statistical method. While LDA remains the most often used natural language model [57,58], by pairing LDA with the HJ-Biplot, we revealed hidden micro-trends and mathematically isolated critical research gaps in BCC research. Since they indicated the strongest associations among variables, reflecting higher correlation levels, clustered heatmaps (Figure 5, Figure 6 and Figure 7), where high-intensity color nodes validated the theme output.

It is valuable to benchmark the identified temporal changes in BCC research against broader bibliometric trends in oncology and dermatology. The transition we observed toward advanced diagnostics and AI aligns with a macro-level surge in digital dermatology [59], though BCC historically trails melanoma in AI dataset representation [60,61]. Additionally, the prominent rise in the Hedgehog signaling pathway in our dataset reflects oncology’s broader evolution toward targeted molecular therapies over the last two decades [62]. Lastly, the continuous focus on surgical outcomes and therapeutic clearance mirrors established bibliometric trends in dermatologic surgery, where localized tissue-sparing modalities remain the academic gold standard [63]. Future comparative bibliometric studies directly contrasting BCC against other cutaneous malignancies could further elucidate these cross-disciplinary knowledge transfers.

## 5. Conclusions

In summary, this study provides insights that may influence both current treatment and underexplored medical research on BCC. This research has implications for dermatologists highlighting the rapid transition toward reflectance confocal microscopy (RCM) and dermoscopy as a standard of care to optimize pre-surgical borders and minimize unnecessary invasive excisions, for policy makers providing an objective roadmap of global research allocation (AI diagnostics) vs. where critical, under-funded structural gaps remain (long-term post-surgical quality of life (QoL) metrics), and for cancer researchers revealing the specific sub-thematic boundaries where for instance Hedgehog signaling resistance mechanisms require deeper bench science. Our comprehensive bibliometric analysis, based on an unbiased machine learning approach, revealed the historical and contemporary facets of BCC research and highlighted emerging directions and trends.

## Figures and Tables

**Table 1:** Comprehensive overview of data characteristics and publication metrics on BCC research between 1973 and 2023.

Description	Result
**Main information about data**	
Timespan	1973:2023
Sources (journals)	3625
Documents	23,680
Annual growth rate %	4.73
Document average age	15.2
Average citations per doc	32.03
**Document contents:**	
Keywords Plus	44,653
Author’s keywords	22,854
**Authors:**	
Authors	77,002
Authors of single-authored docs	1442
**Authors collaboration**	
Single-authored docs	1725
Co-authors per doc	5.39
International co-authorships (%) ^a^	14.24
**Document types:**	
Article	19,770
Review	3910

^a^ International co-authorship: When at least one co-author was based in a country different from the corresponding author’s institution. Retrieved from the Scopus database.

**Table 2:** Top 30 scientific journals for research on BCC based on 22,902 articles published between 1973 and 2023.

Source	NP	TC	h_Index	PY_Start
*Journal of the American Academy of Dermatology*	570	37,136	97	1980
*British Journal of Dermatology*	523	30,668	90	1973
*Dermatological Surgery*	475	13,455	59	1995
*Journal of Cutaneous Pathology*	345	9208	49	1974
*American Journal of Dermatopathology*	340	8074	49	1980
*Journal of the European Academy of Dermatology and Venereology*	301	9066	50	1996
*Journal of Investigative Dermatology*	273	17,334	71	1973
*International Journal of Dermatology*	243	5660	37	1977
*Archives of Dermatology*	185	15,521	72	1973
*Korean Journal of Dermatology*	175	371	9	1974
*Dermatology*	169	3330	32	1973
*Acta Dermato-Venereologica*	159	4115	37	1973
*The Journal of Dermatologic Surgery and Oncology*	158	5261	33	1975
*Journal of Dermatology*	151	2502	27	1979
*Anais Brasileiros De Dermatologia*	150	2267	25	1994
*Hautarzt*	146	1221	17	1973
*Cancers*	145	3367	25	2010
*Australasian Journal of Dermatology*	142	2845	29	1974
*Cancer*	138	11,873	65	1973
*Clinical And Experimental Dermatology*	131	6672	28	1981
*Archives of Dermatological Research*	130	2442	26	1974
*European Journal of Dermatology*	129	3143	30	1993
*Cutis*	125	1121	18	1973
*Annals of Plastic Surgery*	124	1757	22	1978
*Cancer Research*	123	12,717	66	1973
*Dermatology Online Journal*	122	795	12	2000
*Actas Dermo-Sifiliograficas*	119	1001	17	1973
*Photodiagnosis and Photodynamic Therapy*	107	5513	25	2004
*Journal of Drugs in Dermatology*	106	1254	20	2008

Retrieved from the Scopus database. Abbreviations: TC, total citations; NP, number of publications; and PY_start, year of publication start.

**Table 3:** Top ten most prolific authors in BCC from 22,902 articles published between 1973 and 2023.

Author	NP	TC	h-Index	PY_Start
Argenziano G	85	4195	33	1974
Han J	72	4007	32	2004
Lear Jt	55	2745	32	1996
Pellacani G	91	2994	31	2004
Green Ac	72	3020	30	1985
Karagas Mr	46	3660	30	1992
Marghoob Aa	54	2142	30	1995
Stockfleth E	53	3075	30	1990
Zalaudek I	66	3704	30	2003
Dummer R	73	4014	29	1994

Abbreviations: TC, total citations; NP, number of publications; and PY_start = year of publication start. Retrieved from the Scopus database.

**Table 4:** Top 30 countries on BCC research. Citation and article statistics by country according to the analysis of 23,680 articles published between 1973 and 2023.

Country	NP	TC	Average Article Citations
The USA	6688	210,348	49.5
Germany	1580	33,006	32.5
The United Kingdom	1360	42,624	48.7
Italy	1342	26,339	27
Japan	1145	11,107	19.9
China	1009	13,563	14.3
France	810	21,102	41.5
Australia	786	24,218	43.2
Spain	669	11,821	23.5
India	573	4209	9.1
Turkey	548	3606	8
Brazil	532	5753	13.1
South Korea	507	4756	13.9
The Netherlands	497	15,058	41.3
Canada	471	12,833	36.6
Poland	391	2673	9.6
Switzerland	285	9162	51.5
Sweden	269	9460	54.4
Iran	268	2117	8.9
Denmark	263	9749	49.2
Austria	215	6891	46.9
Greece	213	4276	24.9
Belgium	193	4287	32.5
Portugal	187	2117	16.5
Israel	183	4492	35.4
Romania	164	1335	10
Mexico	143	692	6.8
Finland	136	5113	56.2
Czech Republic	115	1465	18.3
Egypt	105	1273	15.3

Retrieved from the Scopus database.

**Table 5:** Topics discovered from 23,680 articles published about BCC between 1973 and 2023. Topic prevalence and top terms.

T	Label	Prevalence (%)	NP	Top_Terms
t_1	Treatment of Patients	5.418	1337	patient, treatment, week, respons, dose, effect, group, trial, receiv, safeti, term, event, efficaci, therapi, long
t_2	Imaging and Diagnostic Methods in Dermatology	5.706	1685	imag, diagnosi, method, diagnost, evalu, detect, specif, clinic, sensit, base, featur, skin, perform, model, accuraci
t_3	Cutaneous Lesions, Melanoma, and Pigmented Tumors	3.996	609	lesion, malign, melanoma, skin, cutan, benign, pigment, keratosi, actin, skin_lesion, malign_melanoma, skin_tumor, melanocyt, ak, common
t_4	Long-Term Studies and Follow-Up in Cancer Therapy	5.35	846	patient, year, group, follow, month, surviv, conclus, method, outcom, median, rang, treat, time, retrospect, rate
t_5	Evidence-Based Dermatology Practices and Guidelines	4.65	758	studi, review, clinic, includ, dermatologi, manag, care, data, practic, dermatologist, health, medic, assess, evid, limit
t_6	Immunotherapy and Immune Response in Cancer	3.776	691	immun, review, recent, hpv, discuss, system, develop, type, therapeut, respons, potenti, agent, current, approach, understand
t_7	Hedgehog Signaling Pathway in Cancer	4.248	1019	pathwai, signal, activ, target, cancer, inhibitor, hedgehog, develop, advanc, hh, vismodegib, drug, gli, signal_pathwai, inhibit
t_8	Genetic Studies and Mutations in Gorlin Syndrome	4.171	1415	syndrom, mutat, gene, multipl, ptch, genet, nbcc, famili, dna, gorlin, variant, nevoid, develop, carcinoma, character
t_9	Clinical Cases and Studies on Specific Skin Tumors	5.456	2048	case, report, present, clinic, diagnosi, year, rare, histopatholog, examin, ulcer, biopsi, literatur, reveal, report_case, featur
t_10	Cancer Risks and Studies in Transplant Recipients	2.986	437	transplant, cancer, develop, malign, immunosuppress, recipi, organ, lung, lymphoma, transplant_recipi, patient, renal, prostat, sarcoma, incid
t_11	Tumors Related to Hair Follicles and Immunohistochemical Markers	5.467	1760	tumor, cell, differenti, posit, stain, pattern, basaloid, epitheli, immunohistochem, neoplasm, hair, neg, featur, antibodi, sebac
t_12	Parotid Gland Tumors and Metastasis Behavior	4.244	909	tumor, local, primari, neck, malign, metastat, metastasi, head, invas, metastas, gland, head_neck, region, node, prognosi
t_13	Smoking and Risk of Skin Cancer	5.113	1078	risk, cancer, increas, ci, associ, factor, control, skin_cancer, skin, compar, ratio, high, popul, melanoma, studi
t_14	Incidence and Epidemiology of Skin Cancers	4.374	726	ag, year, incid, male, femal, rate, diagnos, period, common, ag_year, popul, type, data, hospit, site
t_15	Histological Subtypes and Characteristics of Basal Cell Carcinoma	3.808	422	bcc, subtyp, histolog, nodular, aggress, infiltr, type, superfici, common, background, area, conclus, carcinoma_bcc, head, compar
t_16	Sun Exposure and Skin Cancer Prevention in Childhood	4.806	1088	skin, cancer, skin_cancer, nmsc, exposur, melanoma, radiat, sun, develop, melanoma_skin, factor, prevent, expos, nonmelanoma, squamou
t_17	Inflammatory and Chronic Skin Diseases	2.734	177	diseas, skin, condit, inflammatori, chronic, dermatolog, disord, skin_diseas, infect, psoriasi, includ, cutan, arsen, system, affect
t_18	Protein Expression and Studies on Cutaneous Tumors	6.208	2072	express, cell, tumor, normal, human, protein, tissu, level, role, gene, induc, suggest, breast, growth, investig
t_19	Characterization of Tumors and Immunohistochemical Diagnosis	2.992	112	scc, carcinoma, tumor, squamou, squamou_carcinoma, squamou_scc, bcc_scc, cutan, scc_bcc, bcc_squamou, skin_tumour, mcc, invas, skin, sk
t_20	Photodynamic Therapies and Studies on Skin Cancer	4.438	1408	treatment, therapi, effect, pdt, treat, topic, imiquimod, light, superfici, photodynam, photodynam_therapi, ala, applic, laser, modal
t_21	Mohs Surgery and Histological Clearance of Basal Cell Carcinoma	4.488	1214	excis, recurr, surgeri, surgic, margin, tumor, moh, rate, mm, micrograph, size, section, treatment, micrograph_surgeri, surgic_excis
t_22	Facial Reconstruction and Surgical Techniques for Skin Defects	5.57	1869	flap, reconstruct, defect, eyelid, complic, nasal, techniqu, graft, lower, tissu, facial, function, procedur, case, wound

Topics with the best coherence score or semantic quality based on the LDA model. Abbreviations: t, topic; N, number of publications.

## Data Availability

The data that support the findings of this study are available on request from the corresponding author.

## Abbreviations

The following abbreviations are used in this manuscript:

AI	Artificial Intelligence
HFUS	High-Frequency Ultrasound
LDA	Latent Dirichlet Allocation
RCM	Reflectance Confocal Microscopy
